# Prognosis of patients excluded by the definition of septic shock based on their lactate levels after initial fluid resuscitation: a prospective multi-center observational study

**DOI:** 10.1186/s13054-017-1935-3

**Published:** 2018-02-24

**Authors:** Byuk Sung Ko, Kyuseok Kim, Sung-Hyuk Choi, Gu Hyun Kang, Tae Gun Shin, You Hwan Jo, Seung Mok Ryoo, Jin Ho Beom, Woon Yong Kwon, Kap Su Han, Han Sung Choi, Sung Phil Chung, Gil Joon Suh, Tae Ho Lim, Won Young Kim, for the Korean Shock Society (KoSS) Investigators

**Affiliations:** 1 Department of Emergency Medicine, Hanyang University College of Medicine, Seoul, Korea; 2 Seoul National University Bundang Hospital, Seongnam, Korea; 3 Guro Hospital, Korea University Medical Center, Seoul, Korea; 4 Hallym University College of Medicine, Seoul, Korea; 5 Samsung Medical Center, Sungkyunkwan University School of Medicine, Seoul, Korea; 6 Asan Medical Center, University of Ulsan College of Medicine, Seoul, Korea; 7 Yonsei University College of Medicine, Seoul, Korea; 8 Seoul National University College of Medicine, Seoul, Korea; 9 College of Medicine, Korea University, Seoul, Korea; 10 Kyung Hee University Hospital, Seoul, Korea; 11 Hanyang University College of Medicine, Seoul, Korea; 12 Department of Emergency Medicine, University of Ulsan College of Medicine, Asan Medical Center, 88 Olympic-ro 43-gil, Songpa-gu, Seoul, 05505 Korea

**Keywords:** Lactate, Septic shock, Perfusion, Emergency department

## Abstract

**Background:**

Septic shock can be defined both by the presence of hyperlactatemia and need of vasopressors. Lactate levels should be measured after volume resuscitation (as per the Sepsis-3 definition). However, currently, no studies have evaluated patients who have been excluded by the new criteria for septic shock. The aim of this study was to determine the clinical characteristics and prognosis of these patients, based on their lactate levels after initial fluid resuscitation.

**Methods:**

This observational study was performed using a prospective, multi-center registry of septic shock, with the participation of 10 hospitals in the Korean Shock Society, between October 2015 and February 2017. We compared the 28-day mortality between patients who were excluded from the new definition (defined as lactate level <2 mmol/L after volume resuscitation) and those who were not (≥2 mmol/L after volume resuscitation), from among a cohort of patients with refractory hypotension, and requiring the use of vasopressors. Other outcome variables such as in-hospital mortality, intensive care unit (ICU) stay (days), Sequential Organ Failure Assessment (SOFA) scores and Acute Physiology and Chronic Health Evaluation (APACHE) II scores were also analyzed.

**Results:**

Of 567 patients with refractory hypotension, requiring the use of vasopressors, 435 had elevated lactate levels, while 83 did not have elevated lactate levels (either initially or after volume resuscitation), and 49 (8.2%) had elevated lactate levels initially, which normalized after fluid resuscitation. Thus, these 49 patients were excluded by the new definition of septic shock. These patients, in whom perfusion was restored, demonstrated significantly lower age, platelet count, and initial and subsequent lactate levels (all *p* < 0.01). Similarly, significantly lower 28-day mortality was observed in these patients than in those who had not been excluded (8.2% vs 25.5%, *p* = 0.02). In-hospital mortality and the maximum SOFA score were also significantly lower in the excluded patients group (*p* = 0.03, both).

**Conclusions:**

It seems reasonable for septic shock to be defined by the lactate levels after volume resuscitation. However, owing to the small number of patients in whom lactate levels were improved, further study is warranted.

**Electronic supplementary material:**

The online version of this article (10.1186/s13054-017-1935-3) contains supplementary material, which is available to authorized users.

## Background

Sepsis and septic shock are serious public health problems, and have accounted for more than USD 20 billion of all hospital costs in the USA in 2011 [1]. The incidence of sepsis continues to increase owing to the growing population of aging adults with chronic diseases, and is associated with higher mortality rates despite advances made in the understanding and management of it [2–4]. Recently, the Society for Critical Care Medicine and European Society of Intensive Care Medicine put together a taskforce, with the aim of developing a new definition for sepsis and septic shock (Sepsis-3). Based on this, septic shock was defined as a subset of sepsis that includes underlying circulatory cellular and metabolic abnormalities. Based on Sepsis-3, septic shock was clinically defined as sepsis associated with persisting hypotension, requiring the use of vasopressors to maintain mean arterial pressure (MAP) ≥65 mmHg, and serum lactate >2 mmol/L (18 mg/dL), despite adequate volume resuscitation [5]. It is important for clinicians to realize that the definition of septic shock should be based on the lactate levels after volume resuscitation, as per the Sepsis-3 definition, and not on the lactate levels at the time of the recognition of septic shock. Although there is currently no literature based on controlled data to support the definition of septic shock based on initial fluid resuscitation, it may be possible to follow the existing definition as the average pre-randomization volumes of fluid, used in recent interventional studies, such as the ProCESS, ProMISE, and ARISE trials, were approximately 30 mL/kg or 2 L [6–8]. No studies to date have evaluated the prognoses of patients with initial lactate elevation >2 mmol/L, which then reduces to < 2 mmol/L, after fluid resuscitation – these are the patients who are excluded from the definition of septic shock, based on the Sepsis-3 definition.

To address this issue, we aimed to determine the clinical characteristics and prognosis of those patients who were excluded from the definition of septic shock based on their lactate levels after initial fluid resuscitation. For this, we used data from a prospective, multi-center registry of patients with septic shock, as part of which serum lactate levels are routinely measured at the time of the recognition of the shock and after the fluid challenge

## Methods

### Study design and population

This prospective, multi-center, observational study was conducted in 10 Korean university affiliated hospital emergency departments (ED) using data from the Korean Shock Society (KoSS) septic shock registry, from October 2015 to February 2017. The KoSS is a collaborative research network that is involved in investigating and improving the quality of diagnosis and management of sepsis, and was established in 2013. Since October 2015, investigators who are part of the KoSS have been prospectively collecting data pertaining to patients with septic shock at 10 teaching hospitals across South Korea (Additional file 1) [9]. In this septic shock registry, patients who were aged ≥ 19 years and met the inclusion criteria were enrolled. The inclusion criteria included evidence of refractory hypotension or hypoperfusion in patients with suspected or confirmed infection [6–8]. Hypotension was defined as a systolic blood pressure (SBP) <90 mmHg, MAP <70 mmHg, or SBP decrease >40 mmHg [10]. Refractory hypotension was defined as persistent hypotension despite the administration of fluid challenge (20–30 mL/kg) or of crystalloid solution, was administered over 30 minutes. At least 1 L of crystalloid solution was administered to patients with extremely low body weight or as the requirement of vasopressors to maintain SBP ≥90 mmHg or MAP ≥70 mmHg. Serum lactate levels were routinely measured at the time of the recognition of shock and after the delivery of fluid challenge. Hypoperfusion was defined as serum lactate levels ≥4 mmol/L. Patients were excluded from the study if they were in a “Do Not Attempt Resuscitation” state; if they met inclusion criteria 6 hours after arrival in the ED; if they were transferred from other hospitals and did not meet the inclusion criteria on ED arrival; or if they were transferred directly from the ED to other hospitals. The ED septic shock protocol in our country employs the 6-hour septic shock bundle; hence, we excluded patients that met the inclusion criteria 6 hours after arrival in the ED, so as to minimize the effect of different treatments in each hospital. The institutional review board of each institution approved the study protocol and informed consent was obtained before data collection (Additional file 2).

**Fig. 1:**
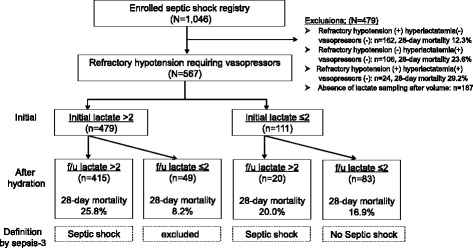
Patient flow diagram. f/u follow up

In this study, patients (enrolled in the septic shock registry) who did not receive vasopressors regardless of lactate levels or those in whom the subsequent lactate levels were not measured were excluded. This population with refractory hypotension, requiring vasopressors, was divided into two groups according to the initial lactate levels (Fig. 1). Each of these groups was further divided based on the subsequent lactate levels after fluid resuscitation. Finally, 28-day mortality in patients in whom the initial lactate levels were > 2 mmol/L and then reduced to < 2 mmol/L after fluid resuscitation, who did not meet the new criteria for septic shock, was compared to that in patients in whom the lactate levels remained > 2 mmol/L.

### Data collection

The case report form of the KoSS septic shock registry, includes standard definitions of 200 variables including clinical characteristics, therapeutic interventions, and outcome of patients with septic shock, and an investigator manual was developed based on a literature review and a consensus of the study investigators. The data are collected via a standardized registry form and entered into a web-based electronic database. Outliers or incorrect values are primarily filtered by this data entry system. In addition, the principal investigator of every site works with a designated local research coordinator, who is responsible for ensuring the accuracy of the data entry and verifying the records. A quality management committee, which consists of emergency physicians, local research coordinators, and investigators from every ED, was established to monitor and review data quality regularly. Members from the committee provide feedback on the results of the quality management process to the research coordinators and investigators, and doubts pertaining to the data are clarified either through the use of the system’s query function or directly through a telephone call.

For our study, demographic and clinical data, including age, gender, medical history, initial vital signs, severity, laboratory values on admission, and interventions were retrieved from the septic shock registry. The primary endpoint of this study was 28-day mortality. The secondary outcomes were in-hospital mortality, ED disposition, and the length of stay in the ICU and in hospital. Severity of septic shock was assessed using the disease severity score. The sequential organ failure assessment (SOFA) score and the maximum SOFA and Acute Physiology and Chronic Health evaluation (APACHE) II scores were evaluated using the worst parameters, within 24 hours after ED arrival [11, 12].

### Statistical analyses

Continuous variables analyzed as the mean ± standard deviation or median with the interquartile range, and absolute or relative frequencies were analyzed for the categorical variables. The Student *t* test or Mann-Whitney *U* test were used to compare the continuous variables, and the chi-square test or the Fisher exact test was used for categorical variables. Comparisons of 28-day mortality and other outcomes in the two groups, based on the recovery of perfusion (lactate level), were performed. A two-sided *P* value ≤0.05 was considered statistically significant. All statistical analyses were performed using SPSS for Windows version 18.0 (SPSS Inc., Chicago, IL, USA).

## Results

### Clinical characteristics

During the study period, 1046 patients were enrolled in the KoSS septic shock registry. Of these, 292 patients who had hyperlactatemia without refractory hypotension were excluded. There were also 187 patients, in whom the subsequent lactate levels after volume resuscitation were not measured, who were also excluded (Fig. 1). Finally, 567 patients were included in our study. Of these, 435 patients had elevated lactate levels (≥2 mmol/L) after fluid resuscitation. There were 83 patients in whom the lactate level was not elevated either initially or after fluid resuscitation. In 49 patients, elevated lactate levels were observed initially but these decreased after fluid resuscitation. The baseline characteristics of 484 patients, based on the Sepsis-3 definition, are outlined in Table 1. The median age of the cohort was 71 years and 57.6% of these patients were male. There was no significant difference in comorbidities between the patients with sepsis in whom perfusion was restored and patients with septic shock as per the Sepsis-3 definition. There was no significant difference between the two groups in the focus of the infection. The rate of positive cultures and the frequency of resistance to initial antibiotics were not significantly different between the groups.

**Table 1:** Baseline characteristics of the study population according to the definition of Sepsis-3

Variables	Overall (*N* = 484)	Sepsis with restored perfusion (*n* = 49)	Sepsis-3 septic shock (*n* = 435)	*p*
Age (years)	71 (60–78)	74 (69–79)	70 (60–78)	0.05
Sex (male)	279 (57.6)	29 (59.2)	250 (57.5)	0.88
Comorbidities				
Hypertension	218 (45.0)	28 (57.1)	190 (43.7)	0.10
Diabetes mellitus	169 (34.9)	14 (28.6)	155 (35.6)	0.35
Cardiac disease	64 (13.2)	6 (12.2)	58 (13.3)	1.00
Cerebrovascular disease	72 (14.9)	10 (20.4)	62 (14.3)	0.29
Chronic lung disease	29 (6.0)	5 (10.2)	24 (5.5)	0.20
Hematologic malignancy	27 (5.6)	3 (6.1)	24 (5.5)	0.75
Metastatic cancer	101 (20.9)	9 (18.4)	92 (21.1)	0.72
Chronic renal disease	42 (8.7)	4 (8.2)	38 (8.7)	1.00
Liver cirrhosis	78 (16.1)	5 (10.2)	73 (16.8)	0.31
Suspected infection focus				
Respiratory infection	153 (31.6)	21 (42.9)	132 (30.3)	0.08
Gastrointestinal	82 (16.9)	6 (12.2)	76 (17.5)	0.43
Hepatobiliary pancreas	113 (23.3)	8 (16.3)	105 (24.1)	0.29
Bone and soft tissue	24 (5.0)	4 (8.2)	20 (4.6)	0.29
Others	14 (2.9)	0 (0.0)	14 (3.2)	0.38
Unknown focus	26 (5.4)	0 (0.0)	26 (6.0)	0.10
Initial vital signs				
Systolic blood pressure (mmHg)	94 (78–122)	108 (78–134)	92 (78–120)	0.07
Diastolic blood pressure (mmHg)	57 (48–69)	59 (50–74)	57 (47–69)	0.36
Respiratory rate (per minute)	21 (20–24)	20 (20–25)	21 (20–24)	0.61
Heart rate (per minute)	110 (95–128)	112 (94–133)	110 (95–128)	0.74
Body temperature (°C)	37.5 (36.5–38.6)	38.0 (36.5–39.3)	37.4 (36.5–38.5)	0.10
Culture positive				
Blood	249 (51.4)	179 (51.7)	59 (51.3)	0.95
Other	225 (46.5)	171 (49.4)	48 (41.7)	0.17
Resistance to initial antibiotics	52 (10.7)	7 (14.3)	45 (10.3)	0.46

Data are shown as median with interquartile range or as number (%)

The median initial and subsequent lactate levels differed significantly, and the platelet count and prothrombin time were also significantly different between the two groups (Table 2). The arterial pH, and the bicarbonate and procalcitonin levels were significantly different (*p* < 0.01, *p* < 0.01, and *p* = 0.02, respectively), but the other laboratory findings did not significantly differ between the two groups.

**Table 2:** Laboratory test findings of the study cohort according to the definition of Sepsis-3

Variables	Overall (N = 484)	Sepsis with restored perfusion (n = 49)	Sepsis-3 septic shock (n = 435)	*p*	Measurements (number)
Initial lactate level (mmol/L)	4.4 (2.8–6.2)	2.7 (2.3–3.8)	4.6 (3.0–6.4)	<0.01	484
Subsequent lactate level (mmol/L)	3.5 (2.2–5.5)	1.6 (1.3–1.8)	3.8 (2.5–6.0)	< 0.01	451
White blood cell count (∙10^3^/L)	10.3 (5.1–17.8)	11.5 (5.1–18.3)	10.2 (5.1–17.8)	0.46	484
Hemoglobin (g/dL)	11.1 (9.4–13.0)	10.7 (9.3–12.9)	11.2 (9.4–13.0)	0.43	484
Hematocrit (%)	33 (28.9–38.7)	32.2 (28.1–39.1)	33.8 (28.9–38.7)	0.55	484
Platelet count (∙10^3^/L)	133 (63–209)	182 (114–288)	127 (62–201)	< 0.01	484
Sodium (mmol/L)	135 (132–139)	135 (132–138)	135 (131–139)	0.50	484
Potassium (mmol/L)	4.1 (3.7–4.6)	4.2 (3.8–4.7)	4.1 (3.6–4.6)	0.22	484
Chloride (mmol/L)	100 (96–104)	100 (95–102)	100 (96–104)	0.33	483
Blood urea nitrogen (mg/dL)	30 (21–45)	28 (18–38)	30 (21–46)	0.07	480
Creatinine (mg/dL)	1.6 (1.1–2.5)	1.4 (0.9–2.3)	1.7 (1.1–2.6)	0.07	480
AST (U/L)	47 (27–119)	36 (24–93)	48 (27–122)	0.15	483
ALT (U/L)	30 (16–73)	24 (13–63)	30 (16–75)	0.14	483
Albumin (g/dL)	2.9 (2.4–3.3)	3.2 (2.7–3.4)	2.9 (2.4–3.3)	0.10	472
Prothrombin time (INR)	1.4 (1.2–1.6)	1.3 (1.1–1.4)	1.4 (1.2–1.6)	0.01	476
C-reactive protein (mg/dL)	13.5 (5.8–21.8)	12.5 (7.0–19.1)	14.0 (5.7–21.9)	0.90	482
Glucose (mg/dL)	135 (102–193)	148 (117–201)	135 (101–192)	0.20	483
Arterial pH	7.42 (7.35–7.47)	7.45 (7.39–7.49)	7.41 (7.34–7.46)	< 0.01	480
PaCO_2_ (mmHg)	28.0 (23.0–33.0)	28.8 (24.6–35.4)	27.8 (23.0–32.5)	0.10	480
PaO_2_ (mmHg)	77.5 (63.9–96.0)	76.0 (63.3–94.3)	78.0 (64.0–96.0)	0.87	480
Bicarbonate (arterial, mmol/L)	17.9 (14.5–21.2)	20.4 (18.0–24.2)	17.7 (14.3–20.7)	< 0.01	480
Procalcitonin (mmol/L)	14.1 (3.0–42.9)	5.3 (0.7–25.2)	14.4 (3.3–45.0)	0.02	401
D-dimer (mcg/mL)	5.0 (2.6–11.6)	3.9 (2.8–9.7)	5.2 (2.6–11.8)	0.54	359
Troponin I or T (ng/mL)	0.06 (0.02–0.27)	0.04 (0.02–0.17)	0.07 (0.02–0.30)	0.21	401

Data are shown as median with interquartile range

*AST* aspartate transaminase, *ALT* alanine transaminase, *BNP* brain natriuretic peptide, *INR* international normalized ratio *PaCO*_*2*_ partial arterial pressure of carbon dioxide

### Outcome variables

We compared the clinical outcomes of the group of patients who were excluded from the new septic shock definition (the excluded group, defined by initial lactate level ≥2 mmol/L; subsequent lactate level <2 mmol/L) with the Sepsis-3 septic shock group (defined by subsequent lactate level ≥2 mmol/L regardless of initial lactate level). The 28-day mortality was significantly lower in the excluded group than in the Sepsis-3 septic shock group (8.2% vs 26.4%, *p* = 0.01) (Table 3). The excluded group also had lower in-hospital mortality than in the Sepsis-3-defined septic shock group (12.2% vs 30.1%, *p* = 0.03). The maximum SOFA scores in the excluded group within 24 hours after ED arrival were significantly lower than in the Sepsis-3 septic shock group (*p* = 0.03). The in-hospital mortality, SOFA score, APACHE II score, and length of ICU stay and hospital stay were not significantly different between the two groups.

**Table 3:** Comparison of outcomes between patients with restored perfusion and those compatible with the Sepsis-3 definition

Outcomes	Sepsis with restored perfusion (*n* = 49)	Sepsis-3 septic shock (*n* = 435)	*p*
Mortality at 28 days	4 (8.2%)	115 (26.4%)	0.01
In-hospital mortality	6 (12.2%)	131 (30.1%)	0.03
SOFA score	6.0 (4.0–8.0)	7.0 (5.0–9.0)	0.09
Maximum SOFA score	9.0 (6.0–10.0)	9.0 (7.0–12.0)	0.03
APACHE II	21.0 (15.0–26.0)	21.0 (16.0–27.0)	0.92
ICU stay (days)	4.0 (3.0–5.3)	5.0 (3.0–9.0)	0.26
Hospital stay (days)	12.0 (7.0–16.0)	14.0 (7.0–26.0)	0.26

Data are shown as median with interquartile range or as number (%)

*SOFA* Sequential Organ Failure Assessment, *APACHE* Acute Physiology and Chronic Health Evaluation, *ICU* intensive care unit.

### Comparison of outcomes according to lactate level, hypotension, and vasopressor requirement

The cohort was divided according to the lactate level, hypotension, and vasopressor requirement. The 28-day mortality in group 1 (patients who were hypotensive after fluids and vasopressor therapy and serum lactate levels >2 mmol/L) was 25.5%. The outcomes of the other groups are summarized in Table 4.

**Table 4:** The 28-day mortality in the study cohort according to lactate level, hypotension, and vasopressor requirement

Cohorts	Mortality at 28 days, number/total number of patients per group (percentage)
Group 1 (hypotensive after fluids and vasopressor therapy and serum lactate levels >2 mmol/L)	111/435 (25.5%)
Group 2 (hypotensive after fluids and vasopressor therapy and serum lactate levels ≤2 mmol/L)	18/132 (13.6%)
Group 3 (hypotensive after fluids and no vasopressors and serum lactate levels >2 mmol/L)	18/77 (23.4%)
Group 4 (serum lactate levels >2 mmol/L and no hypotension after fluids and no vasopressors)	20/82 (24.4%)
Group 5 (serum lactate levels between 2-4 mmol/L and no hypotension before fluids and no vasopressors)	Not applicable
Group 6 (hypotensive after fluids and no vasopressors and serum lactate ≤2 mmol/L)	8/86 (9.3%)

## Discussion

This study demonstrated that excluding patients from the definition of septic shock based on the lactate levels after fluid resuscitation (initial lactate level >2 mmol/L, subsequent lactate level ≤2 mmol/L) may be reasonable, as low 28-day mortality (8.2%) was observed in these patients. The patients in whom perfusion was restored also had low in-hospital mortality and maximum SOFA scores. However, the other outcome variables (SOFA score at inclusion, APACHE II score, ICU stay and hospital stay) were not significantly different between the two groups.

The strength of our study is that it was conducted using data from a prospective, multi-center registry. Data in this registry are centrally reviewed and regularly controlled by research coordinators, for quality control. Furthermore, the original inclusion criteria of the registry were based on the Surviving Sepsis Campaign 2012; therefore, a few patients who were compatible with the Sepsis-3 definition may have been excluded [10]. In addition, in this study, all the subsequent lactate levels were obtained after volume resuscitation, as per the protocol, within 6 hours after ED arrival. To the best of our knowledge, this is the first study to examine the prognosis of patients who were excluded from the definition of septic shock, based on their lactate levels after initial fluid resuscitation.

The International Guidelines for Management of Severe Sepsis and Septic Shock recommended the use of bundles for the treatment of sepsis [10]. It was recommended that lactate levels be measured within 3 hours after admission to the ED, and re-measured within 6 hours [10, 13]. Current guidelines also recommend targeting resuscitation to normalize lactate levels, and suggest that lactate levels are a useful predictor of various diseases [14–17]. Based on this, lactate measurement for the early recognition of hypoperfusion and cryptogenic shock has been widely used [8, 10]. Levy et al. reported that the mortality was high in patients with both hypotension requiring the use of vasopressors and lactate levels ≥4 mmol/L (46.1%), while 36.7% mortality was observed in patients with hypotension alone [18]. However, the lactate levels in the previous study were different from those in our study, and it is not clear if they were measured initially or after volume resuscitation. In our study, 28-day mortality in the patients with restored perfusion was significantly lower compared to patients with Sepsis-3 septic shock. It is suggested that the patients in the Sepsis-3 septic shock group had higher mortality due to hypoperfusion and inadequate resuscitation. Initial higher systolic blood pressure and lower lactate in patients in the restored perfusion group might explain our results. It is also possible that mortality in the excluded group was lower, reflecting the small number of seriously ill patients, based on the significantly lower maximum SOFA score. According to Sepsis-3, the clinical diagnostic criteria for septic shock are based on lactate levels after volume resuscitation, whereas treatment guidelines recommend the use of initial lactate levels for the identification of cryptic shock. However, no studies to date have investigated the impact of excluding patients in whom perfusion is restored following sepsis. In the Sepsis-3 septic shock group, in-hospital mortality was significantly higher in patients with fluid-resistant hypotension requiring vasopressors and hyperlactatemia (≥2 mmol/L) compared to those who had hypotension alone (42.3% vs 30.1%) [19]. However, this comparison was conducted using the lactate levels after volume resuscitation, and not the initial values. Therefore, our study advocates the exclusion of patients, in whom perfusion was restored following sepsis, from the Sepsis-3 definition.

This study had several limitations. The main limitation involves the observation that e mortality rates in the excluded patients were lower than those in the patients with initially normal lactate levels, raising concerns about the extent of bias in the results. Although difficult to explain, it may be attributed to the small number of patients in whom perfusion was restored. Second, the 187 patients who were lost to follow up for measurement of lactate levels may also preclude strong conclusions. Third, several patients were excluded due to the lack of informed consent. In addition, we focused on patients with early septic shock in the ED, and not in the ICU, which might have led to selection bias. Even though our study used data from a prospective, multi-center registry, some laboratory variables were not obtained in all patients. However, these laboratory variables were not of interest to us, and did not affect our main results. In addition, because this was a multi-center study, the enrollment periods and case volumes varied by hospital, and institutional characteristics were not adjusted for the analysis. Although our patients underwent protocol-driven septic shock management, it could have affected our results.

## Conclusions

The 28-day mortality in patients who were excluded from the definition of septic shock based on lactate levels after fluid resuscitation was very low (8.2%), which suggests that this exclusion seems appropriate. Our result might help clinicians make a consistent diagnosis of septic shock and to unify communication in both the clinical and research settings. However, owing to the small number of patients in whom lactate levels were improved, further study is warranted.

## Additional files

Additional file 1: Case report form of the KoSS (Korean Shock Society) septic shock registry. (PPTX 600 kb)

Additional file 2: Names of all ethical bodies/institutional review board of all institution that approved your study in the various centers involved. (DOCX 13 kb)

## Abbreviations

APACHE: Acute physiology and chronic health evaluation
ED: Emergency department
ICU: Intensive care unit
KoSS: Korean Shock Society
MAP: Mean arterial pressure
SBP: Systolic blood pressure
SOFA: Sequential organ failure assessment
