# Supplementary figures and images for: Prognosis of patients excluded by the definition of septic shock based on their lactate levels after initial fluid resuscitation: a prospective multi-center observational study
Source: Crit Care. 2018 Feb 24;22:47. doi: 10.1186/s13054-017-1935-3 (PMC6389162; doi:10.1186/s13054-017-1935-3)

## Slide 1
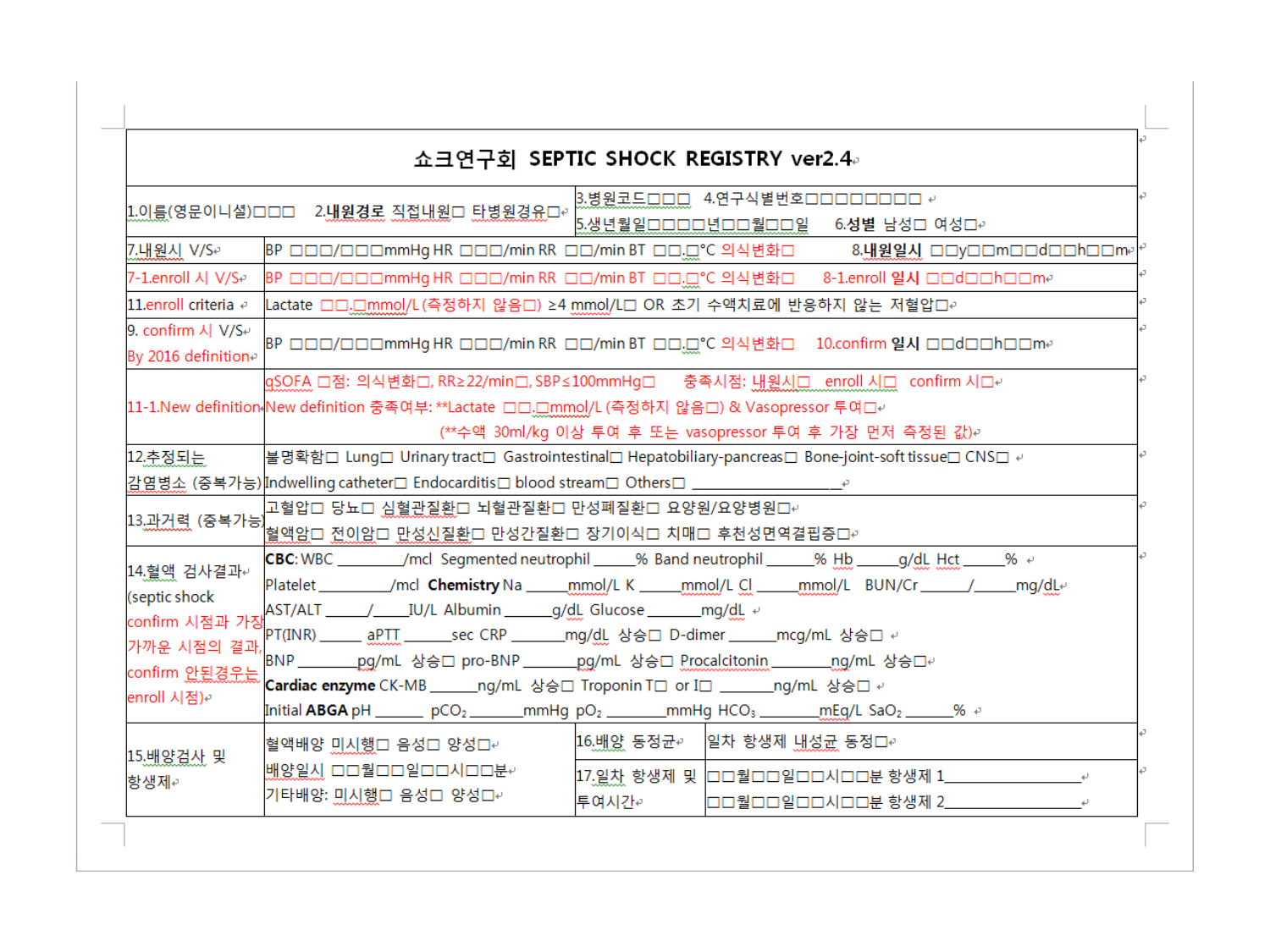

## Slide 2
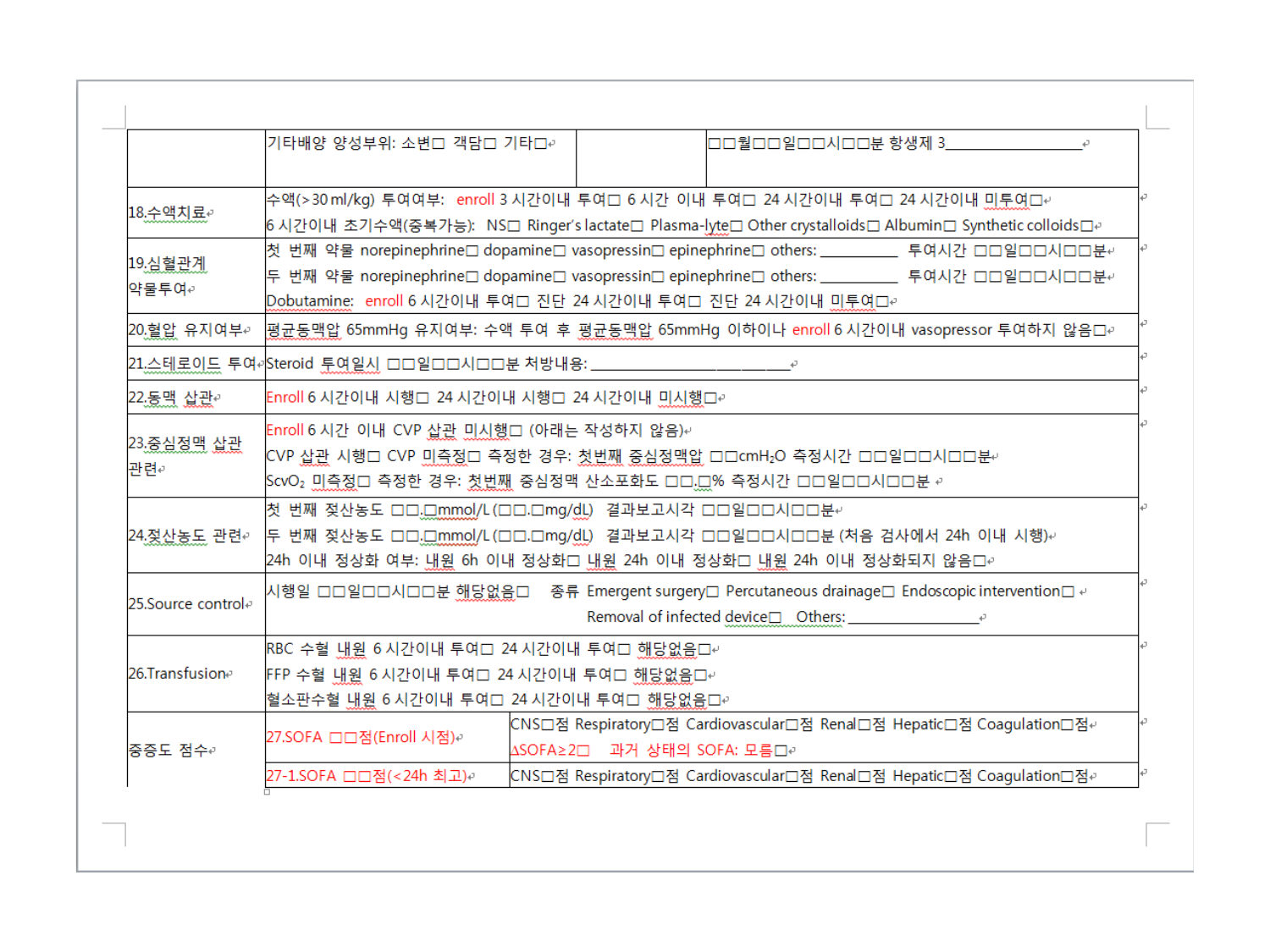

## Slide 3
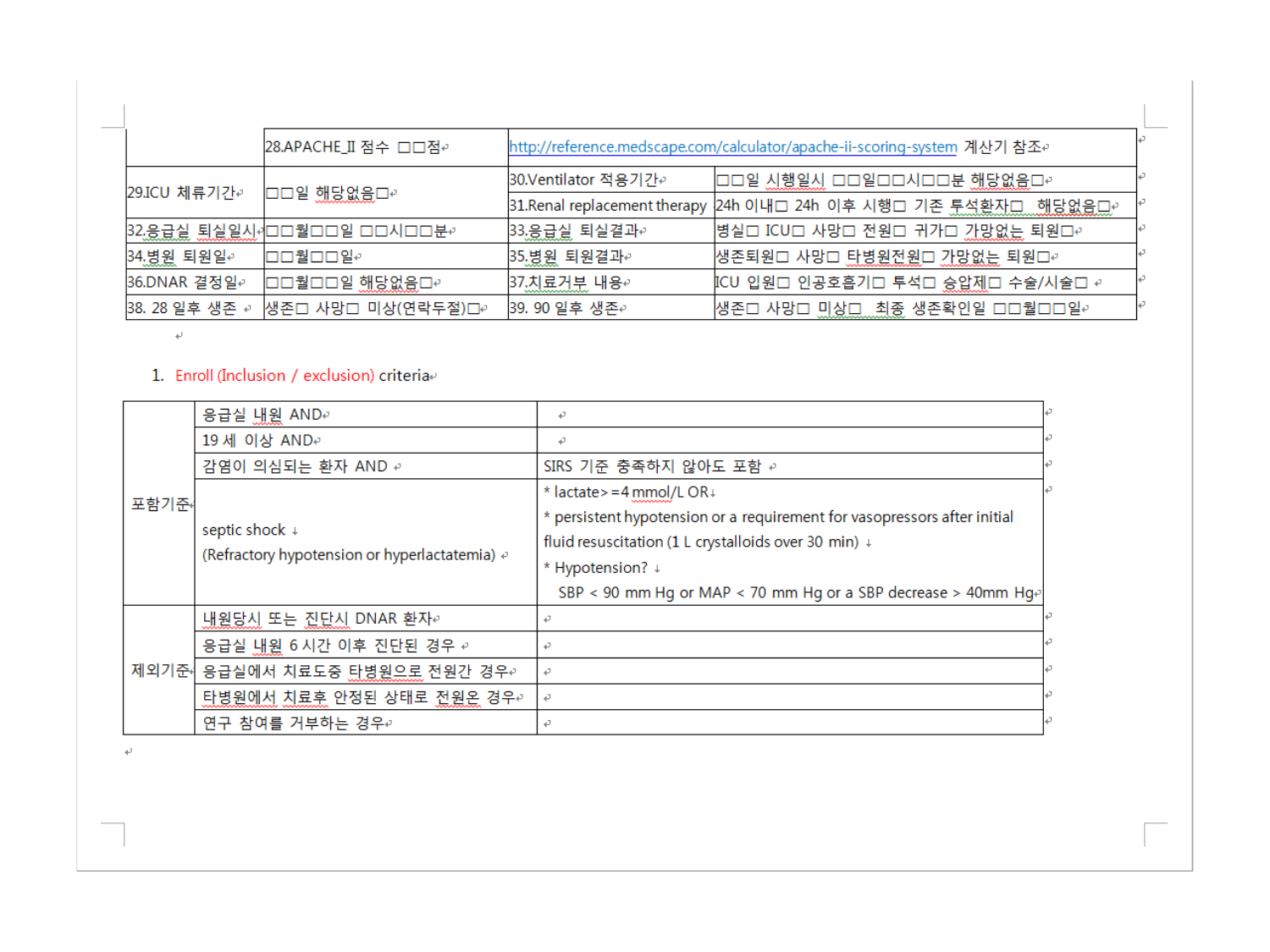

Supplement: Supplementary file 1 — Case report form of the KoSS (Korean Shock Society) septic shock registry. (PPTX 600 kb) [file 13054_2017_1935_MOESM1_ESM.pptx]
