# Supplementary material for: Prognosis of patients excluded by the definition of septic shock based on their lactate levels after initial fluid resuscitation: a prospective multi-center observational study
Source: Crit Care. 2018 Feb 24;22:47. doi: 10.1186/s13054-017-1935-3 (PMC6389162; doi:10.1186/s13054-017-1935-3)
Supplement: Supplementary file 2 — Names of all ethical bodies/institutional review board of all institution that approved your study in the various centers involved. (DOCX 13 kb) [file 13054_2017_1935_MOESM2_ESM.docx]

The research ethics committee of Hallym University Kangnam Sacred Heart Hospital, The research ethics committee of Gangnam Severance Hospital, The research ethics committee of Kyungpook National University Hospital, The research ethics committee of KyungHee University Medical Center, The research ethics committee of Korea University Guro Hospital, The research ethics committee of Korea University Anam Hospital, The research ethics committee of Seoul National University Boramae Medical Center, The research ethics committee of Bucheon ST. Mary’s Hospital, The research ethics committee of Seoul National University Bundang Hospital, The research ethics committee of Samsung Medical Center, The research ethics committee of Seoul National University Hospital, The research ethics committee of Asan Medical Center, The research ethics committee of Severance Hospital, The research ethics committee of Ulsan University Hospital, The research ethics committee of Inje University Ilsan Paik Hospital, The research ethics committee of Chonnam National University Hospital, The research ethics committee of Chonbuk National University Hospital, The research ethics committee of Jeju National University Hospital, The research ethics committee of Chosun University Hospital, Hallym University Sacred Heart Hospital, Hanyang University Seoul Hospital.
